# Self-perceived workplace discrimination and mental health among immigrant workers in Italy: a cross-sectional study

**DOI:** 10.1186/s12888-021-03077-6

**Published:** 2021-02-09

**Authors:** Anteo Di Napoli, Alessandra Rossi, Francesca Baralla, Martina Ventura, Rosaria Gatta, Monica Perez, Marco Sarchiapone, Concetta Mirisola, Alessio Petrelli

**Affiliations:** 1 National Institute for Health, Migration and Poverty (INMP), Epidemiology Unit, Via di San Gallicano 25/a, 00153 Rome, Italy; 2 Department of Medicine and Health Sciences, University of Molise, 86100 Campobasso, Italy; 3 Médecins Sans Frontières (MSF), Via Magenta 5, 00185 Rome, Italy; 4 National Institute of Statistics (Istat), Viale Liegi 13, 00198 Rome, Italy

**Keywords:** Immigrant, Workplace, Mental health status, Discrimination, Mediation analysis

## Abstract

**Background:**

The process of immigration is associated with poor mental and physical health. While the workplace represents an important context of social integration, previous studies evaluating the effect of discrimination experienced in the workplace found worse mental health status among immigrants. The aim of this study was to investigate whether self-perceived workplace discrimination has any role in the mental health status of immigrants living and working in Italy, evaluating the contribution of other personal experiences, such as loneliness and life satisfaction.

**Methods:**

A cross-sectional study was conducted on a sample of 12,408 immigrants (aged 15–64) living and working in Italy. Data were derived from the first national survey on immigrants carried out by the Italian National Institute of Statistics (Istat). Mental health status was measured through the Mental Component Summary (MCS) of the SF-12 questionnaire. A linear multivariate linear regression was carried out to evaluate the association between mental health status, self-perceived workplace discrimination, and sociodemographic factors; path analysis was used to quantify the mediation effect of self-perceived loneliness, level of life satisfaction, and the Physical Component Summary (PCS).

**Results:**

Mental health status was inversely associated (*p* < 0.001) with self-perceived workplace discrimination (β:-1.737), self-perceived loneliness (β:-2.653), and physical health status (β:-0.089); it was directly associated with level of life satisfaction (β:1.122). As confirmed by the path analysis, the effect of self-perceived workplace discrimination on MCS was mediated by the other factors considered: self-perceived loneliness (11.9%), level of life satisfaction (20.7%), and physical health status (3.9%).

**Conclusions:**

Our study suggests that self-perceived workplace discrimination is associated with worse mental health status in immigrant workers through personal experiences in the workplace and explains the effect of the exposure to workplace discrimination on immigrants’ psychological well-being. Our findings suggest that an overall public health response is needed to facilitate the social integration of immigrants and their access to health services, particularly those services that address mental health issues.

**Supplementary Information:**

The online version contains supplementary material available at 10.1186/s12888-021-03077-6.

## Background

The immigration process and the marginalization related to truncated social support networks result in challenging psychological adjustments and can cause excess mental health complications in immigrant populations [1]. Stressful experiences may result in schizophrenia, psychological distress, depression, and anxiety, as well as in post-traumatic stress disorder and suicidal ideation [2–5].

Social science and medical research in the past two decades, particularly that conducted in North America, has extensively examined the health patterns of immigrants, who tend to be healthier than the native-born population at the time of their arrival in the country. This so-called “healthy migrant effect”, an advantage that tends to be lost over time, is probably the consequence of the poor socioeconomic conditions experienced by immigrants in the host country [6, 7]. In particular, immigrants who experience discrimination or unfair treatment in their host country are more likely to experience a decline in self-reported health status, showing a clear inverse socioeconomic gradient with respect to increasing levels of feelings of sadness, depression, and loneliness [6].

Migration to other countries may be due to war or poverty but may also be motivated by the aspiration to a better life. A fulfilling job can represent a key element in the process of integration in the host society [8]; it can help promote economic independence, planning for the future, meeting members of the host society, learning the language of the host country, restoring self-esteem, and encouraging self-reliance [9]. Work is a relevant dimension of the social gradient in health, being one of psychosocial domains that influence lifetime health conditions [10].

In the case of immigrant workers, current evidence shows that the work organization and employment conditions they face are dangerous to their health. Indeed, immigrant workers are more likely to accept jobs that native workers are reluctant to perform, the so-called 3Ds (dangerous, dirty, degrading) [11]. They are over-represented in precarious, informal jobs, often have no kind of protection or social safety net, suffer from stronger internal competition and discrimination, earn poverty wages, and experience more serious abuse and exploitation in the workplace than do natives [11–13].

In the work environment, discrimination usually occurs when the actions of an employer, supervisor, or co-worker deny individuals or groups of people the equality of treatment they may wish [14]. The workplace provides opportunities for stereotyping, prejudice, and discrimination [15], in particular if it is systemic race-based discrimination [16].

The literature reports that exposure to discrimination is widely understood as a social determinant of psychophysical health, as well as a contributing factor to health inequities between social groups [17–19].

Strong associations between perceived racial discrimination and negative mental health outcomes such as depression and anxiety, psychological distress, and a decline in general well-being (e.g., self-esteem, life-satisfaction, quality of life) have been found in different countries and culture s[17, 20–22]. Individuals who report higher levels or more severe forms of discrimination are exposed to a higher risk of poor health than those who experience discrimination less frequently [12, 23–25].

However, despite the consistency of findings investigating the association between perceived racial discrimination and poor health, research has not adequately addressed the mechanisms and processes by which perceived racial/ethnic discrimination might adversely affect health. Mental health status among immigrants can also be affected by some personal experiences, such as perceived loneliness and life satisfaction. Loneliness has also been documented as relevant to perceived social isolation processes and as a strong predictor of other mental health problems as well as of physical health conditions, particularly in this group of often vulnerable individuals [26].

According to the model proposed by Pascoe & Smart Richman [27], discriminatory experiences may affect health through three pathways: directly, partially mediated through stress responses to a discriminatory event, or through health risk behaviors that may emerge as possible coping mechanisms when discrimination is experienced.

Due to the social and economic crisis that began in 2008, the scarce resources of the European (and specifically of the Italian) labour market have given rise to concerns among the native population that migrants are taking jobs away from them, thereby increasing competition, discrimination, and inequality in the workplace [28, 29]. This climate of xenophobia and discrimination has impacted the lives of immigrants since they are the most deprived workers [11]. A study based on a representative sample of all people residing in Italy found that the mental health status of both Italians and immigrants worsened between 2005 and 2013 (just before and after the global economic crisis), supporting the hypothesis that the worsening of socioeconomic conditions observed during this period could have contributed to mental health decline [30].

In Italy, the number of resident immigrants has doubled, from 2.4 million people (4.1% of the resident population) in 2005 to 5.3 million (8.7%) in 2019 [31].

Given this considerable increase, insight into the health status and quality of life of immigrants in Italy has become essential.

There are about 2.4 million immigrant workers employed in Italy (10.6% of the total workforce), almost 90% of whom are employed in the economic sectors “Other collective and personal services” (36.6%, including caregivers, domestic workers, babysitters, home care services operators), “Hotels and restaurants” (17.9%), “Agriculture” (17.9%) and “Construction” (17.2%). About 80% of immigrants in Italy, especially those from non-EU countries, work as manual workers. Overall, 86.5% of employed immigrants in Italy have an unskilled job, 26% more than native-born workers (the average of OECD countries is 65% for immigrants, with a 10% gap with natives). Moreover, 46.0% of non-EU workers and 50.8% of EU workers declare a high level of satisfaction, compared to 57.5% of Italian workers [32]. Immigrants also present higher occupational injury risk than do Italian workers [33, 34].

Although a previous study found that self-perceived workplace discrimination was more likely among immigrants than among Italians [35], there are still few data on how discrimination affects the mental health of the immigrant workforce in Southern Europe [12, 36].

## Methods

### Aim

The aim of our study was to evaluate whether self-perceived workplace discrimination has any role in the mental health status of immigrants living and working in Italy, quantifying the contribution of loneliness, life satisfaction, and perceived physical health as mediators of this relationship.

### Study design and participants

A cross-sectional study was conducted on a sample of 12,408 immigrants aged 15–64 years, residing in Italy and employed at the time of the survey or were employed in Italy at some time prior to the survey. Immigrants with no history of employment in Italy were excluded.

Data were obtained from the first unique national survey – “Social Conditions and Integration of Foreign Citizens” (SCIF) – conducted in 2011–2012 by the Italian National Institute of Statistics (Istat) [37, 38]. The SCIF survey covered many items concerning the living conditions and social integration of immigrants in Italy. In particular, the SCIF survey collected information on socioeconomic status, migratory routes, work history, physical and mental health status, and self-perceived discrimination in the workplace. SCIF also collected and analysed other factors potentially influencing mental health status, such as self-perceived physical health status, self-perceived loneliness, and self-perceived level of life satisfaction. Information on each family member was collected through face-to-face interviews conducted by means a Computer Assisted Personal Interviewing (CAPI) technique, conducted in the family home by interviewers trained by Istat. To facilitate communication during the interview and to ensure a greater level of understanding of the questions, the questionnaire was translated into 10 languages. This survey was part of the activities included in the National Statistical Programme approved by the Italian Presidency of the Council of Ministers. The selected families received a letter from Istat explaining the purposes of the survey and how it would be conducted. They were also reassured about confidentiality and protection of personal data. Except for some sensitive information specified in the informative letter, the response to the survey was mandatory by law and formal consent to participate was therefore not required [37].

The inclusion criteria was for a household to have at least one resident foreign citizen: The SCIF survey involved a total of 20,379 individuals with foreign citizenship, 4251 individuals with Italian citizenship from birth (nationals), and 696 nationals by acquisition. The nationals, not being the subject of the survey, were investigated only for the information needed to reconstruct the family composition so as to have a complete data set also for mixed families (made up of Italians and foreigners). The survey did not include temporary migrants. The survey considered a sample of 9553 families with at least one foreign citizen residing in one of 833 Italian municipalities. The foreign population to which the estimates refer was calculated starting from resident foreigners in the 2011 census. The number of families extracted was three times higher than the theoretical sample size so as to allow the creation of quatrains of families to be used to replace untraceable families, i.e. those who were untraceable to participate in the survey. A corrector was introduced in the calculation of the carry coefficients to the universe, under the assumption that the responders’ behaviour was similar to that of untraceable families. Italian municipalities were divided into two subsets on the basis of the resident foreign population: the set of self-representative municipalities, made up of the municipalities with the largest demographic size, and the set of municipalities that were not self-representative, made up of the remaining municipalities. Within the self-representative municipalities, each municipality was considered as a separate layer, and cluster sampling was adopted. The primary sampling units were systematically extracted from the Registry Office of the municipality itself. Within the not self-representative municipalities, a two-stage design with stratification of the primary units was adopted: the primary units were the municipalities, the secondary units were the families. The not self-representative municipalities were selected with probabilities proportional to their demographic size and without re-entry, while families were extracted with equal probabilities and without re-entry. The self-representative municipalities were included with certainty in the sample; for the not self-representative municipalities, three sampling municipalities were extracted within each layer with probability proportional to the demographic size to guarantee the coverage and the representativeness of the resident foreigners. In order to ensure the statistical representativity of the 15 principal citizenships in Italy, in terms of their numerical consistency, a balanced sampling for each citizenship was selected. The sampling list was the archive of Italian municipalities, which contains the number of foreign residents by sex and citizenship for each municipality [38]. The Italian metropolitan cities are Turin, Milan, Venice, Genoa, Bologna, Florence, Rome, Naples, Bari, Palermo, Catania, and Cagliari.

### Measures

#### Mental component summary (MCS) and physical component summary (PCS)

Mental and physical health status were calculated on the basis of the validated Italian version of the SF-12, a subset of the larger SF-36 questionnaire [39, 40]. The SF-12 incorporates two dimensions: Physical Component Summary (PCS) and Mental Component Summary (MCS). The MCS includes vitality, social functioning, emotional role, and mental health; the PCS domains include general health, physical status and functioning, and body pain. Both MCS and PCS are indexes with a score ranging from 0 (the worst condition) to 100 (the best condition).

MCS was the outcome variable of the present study.

#### Self-perceived discrimination in the workplace (S-PDW)

Information was obtained by asking, “During your stay in Italy, have you ever experienced discrimination or any prejudices in the workplace?” (yes vs no was considered as the reference category). The SCIF collected information solely on discrimination among adults in the workplace.

#### Self-perceived loneliness (S-PL)

The question “Do you feel lonely in Italy?” was used to assess immigrants’ level of loneliness in the host country. We dichotomized the four possible alternatives: subjects who responded “very much/a fair amount” were considered to have self-perceived loneliness, while those who answered “little/not at all” were considered not to have self-perceived loneliness (reference category).

#### Life satisfaction (LS)

Information about immigrants’ level of life satisfaction was assessed through the question, “On a scale of one to ten, how satisfied are you with your life right now?”

#### Sociodemographic factors

Based on previous studies [8, 20], we also considered some factors that may influence the association between S-PWD and MCS. We dichotomized the variables education level, age, and length of stay. In particular, we analysed the following variables: age (two categories: 15–39 and 40–64), sex, education level (high: ≥ 11 years of schooling, or medium/low: up to 11 years of schooling), employment status (currently employed or formerly employed), area of origin (Europe, North Africa, Sub-Saharan Africa, Central-western Asia, Eastern Asia/ Pacific, the Americas), length of stay in Italy (≤ 9 or ≥ 10 years). Information about age and length of stay in Italy were dichotomized, using the median value of their distribution as a cut-off.

### Statistical analysis

We performed the Wilcoxon-Mann-Whitney and Kruskal-Wallis tests to evaluate the differences in the distribution of MCS score by the characteristics of the study population. We tested the assumption of a linear relationship between PCS and MCS, performing a linear univariate regression model and calculating a Pearson correlation coefficient. The regression coefficient β was equal to − 0.026 (Table 2), describing a weak but statistically significant inverse linear association. The Pearson correlation coefficient between PCS and MCS, showed a significant (*p* = 0.02) negative linear correlation (coefficient:-0.021 (*p-*value = 0.022). We evaluated the association between immigrants’ S-PWD and MCS by calculating regression coefficients (β) through univariate and multivariate linear regression models, taking into account sociodemographic and self-perceived individual factors that could affect the association considered.

**Fig. 1:**
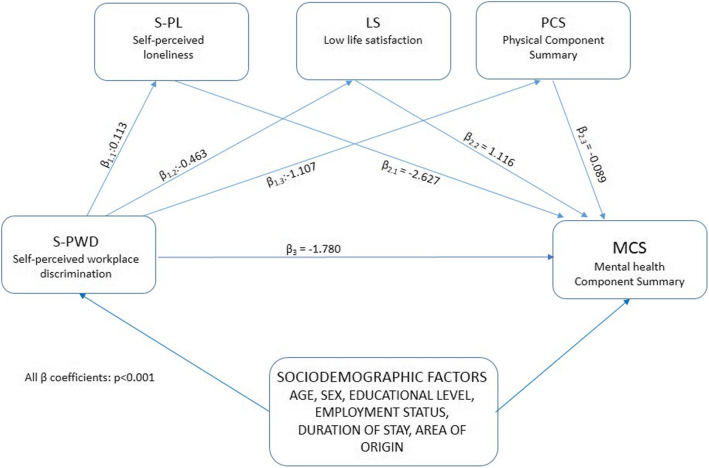
Graphic display of the conceptual path analysis model

Additionally, in order to quantify the mediation effect of self-perceived loneliness, level of life satisfaction, and Physical Component Summary (PCS), a path analysis was performed, as shown in Fig. 1. This approach allowed for the decomposition of the total effect of S-PWD on MCS into direct effects (independent of all mediating factors and confounders) and indirect effects, through each of the mediating factors (S-PL, LS and PCS) [41]. The arrows in Fig. 1 represent regression equations used to assess mediating factors. The regression coefficients were estimated through a path analysis adjusted for all the confounders (age, sex, education level, employment status, length of stay, area of origin) [42]. The direct relationship between exposure factor (S-PWD) and outcome (MCS) was estimated by the coefficient β_3_, while their indirect relationship was decomposed into the coefficients β_1,i_ estimating the connection between S-PWD and each mediating factor (S-PL, LS, PCS) and into the coefficients β_2,i_estimating the connection between each mediating factor (S-PL, LS, PCS) and MCS, where *i* represents the three mediating factors (i = 1,2,3). The indirect effects were then calculated by multiplying the coefficients estimated (β_1,i_* β_2,i_). The output of the path analysis is shown in Additional file 1.

We also calculated the proportions of the direct and indirect effects of the S-PWD out of the total effect on the MCS. The model was adjusted for sociodemographic factors (age, sex, education level, employment status, length of stay, area of origin).

For the multivariate regression model and for the path analysis, we calculated the likelihood-ratio test LR [43], as the difference between − 2 log L from the model with only the intercept and − 2 log L from full model, with the number of degrees of freedom equal to the number of predictors (k = 10)). We obtained LR = 974.01, with the *p*-value (χ^2^_k_ ≥ LR) < 0.001, indicating that the model with all predictors fit significantly better than the model with only the intercept. We also compared the full model with the model with only three determinants (self-perceived workplace discrimination, self-perceived loneliness, and level of life satisfaction), and obtained an LR = 302.39 with 7 degrees of freedom; the *p-*value was (χ^2^_k_ ≥ LR) < 0.-001, indicating that the model with all predictors fit significantly better than the model with three predictors.

Our analyses were performed with SAS 9.3 for descriptive analysis and linear regression models and with STATA 15 for the path analysis.

## Results

In the sample of 12,408 immigrants, individuals were mainly young (mean age 38.9 years, SD10.2), with an average length of stay in Italy equal to 10.7 years (SD 6.4).

Table 1 summarises the characteristics of the study population by MCS score. At the time of the interview, 83.1% of subjects were employed, while 16.9% had a work history in Italy but had lost their job. Most of the immigrants had a middle/low education level (61.3%) and came from Europe (61.3%). Of all interviewed subjects, 17.3% reported self-perceived discrimination in the workplace in Italy, 16.3% declared they felt lonely, and 44.4% felt low satisfaction with their life. Subjects who reported S-PWD had lower MCS mean scores than those who did not (51.4 vs 53.9), as did those who reported S-PL (50.2 vs 54.1) and those who reported having a low LS (51.6 vs 54.9). We found that a unitary increase of level of life satisfaction and PCS were associated with a 1.308 probability of increase and a 0.026 decrease in the MCS score, respectively.

**Table 1:** Individual characteristics and Mental Component Summary (MCS). SCIF survey 2011–2012

		n.	%	MCS score	p-value
				(mean ± SD) / median (IRQ)	
Total sample		12,408	100	(53.5 ± 7.1) / 55.9 (8–2)	–
**Categorical variables**		**n.**	**%**	**MCS score** **(mean ± SD)**	**p-value**
Self-perceived workplace discrimination	No	10,262	82.7	(53.9 ± 6.7)	< 0.0001
	Yes	2,146	17.3	(51.4 ± 8.4)	
Self-perceived loneliness	No	10,385	83.7	(54.1 ± 6.5)	< 0.0001
	Yes	2,023	16.3	(50.2 ± 9.1)	
Length of stay (years)	<=9	6,050	48.8	(53.7 ± 6.8)	0.04
	> = 10	6,358	51.2	(53.3 ± 7.4)	
Age group (years)	15–39	6,746	54.4	(53.9 ± 6.9)	< 0.0001
	40–64	5,662	45.6	(53.0 ± 7.4)	
Sex	Male	6,217	50.1	(53.8 ± 6.8)	< 0.0001
	Female	6,191	49.9	(53.1 ± 7.4)	
Education level	High	4,808	38.7	(53.7 ± 7.2)	< 0.0001
	Middle/Low	7,600	61.3	(53.3 ± 7.1)	
Employment status	Employed	10,316	83.1	(53.8 ± 6.7)	< 0.0001
	Formerly employed	2,092	16.9	(51.8 ± 8.7)	
Area of origin	Europe	7,604	61.3	(53.4 ± 7.1)	< 0.0001
	North Africa	1,526	12.3	(52.8 ± 7.7)	
	Sub-Saharan Africa	711	5.7	(53.3 ± 7.3)	
	Central-western Asia	873	7	(54.3 ± 6.1)	
	East Asia / Pacific	854	6.9	(54.6 ± 6.2)	
	The Americas	840	6.8	(53.3 ± 7.4)	
**Discrete and continuous variables**		**n.**	**mean ± SD /** **median (IRQ)**	**MCS score** **(β coefficient)**	**p-value**
Level of life satisfaction	1 more score value	12,408	7.6 **±** 1.6 / 8 (1)	β = 1.308	< 0.0001
Physical Component Summary (PCS)	1 more PCS value	12,408	54.7 **±** 5.6) / 56 (2)	β = − 0.026	0.022

Among immigrants who self-reported discrimination, 91.4% declared that the reason was due to being a foreigner, 29.2% to their way of speaking Italian, 15.7% to skin colour, 6.8% to religion; in 12.6% of cases the self-reported discrimination was gender-related (data not shown in table).

Table 2 shows the results of the univariate and multivariate linear regression models assessing the association between MCS score, S-PWD, and other factors. The MCS score was inversely associated with the presence of S-PWD (β:-1.737) and of S-PL (β:-2.653), and an unitary increment of PCS score (β:-0.089); it was directly associated with a unitary increment of LS (β:1.122). We also observed a statistically significant association between MCS score and a length of stay in Italy longer than 9 years (β:-0.719), having lost one’s job at the time of the survey (β:-1.502), being a woman (β:-0.726), and being 40–64 years old compared with subjects 15–39 years old (β:-0.618).

**Table 2:** Factors associated with Mental Component Summary (MCS). Crude and adjusted ß coefficients with 95% confidence intervals (CI). SCIF survey 2011–2012

VARIABLES		Crude β	95%CI		p-value	Adjusted β	95%CI		p-value
Self-perceived workplace discrimination	No	0	–	–	–	0	–	–	–
	Yes	−2.496	−2.824	− 2.168	< 0.0001	− 1.737	− 2.051	−1.423	< 0.0001
Self-perceived loneliness	No	0	–	–	–	0	–	–	–
	Yes	−3.898	−4.230	−3.566	< 0.0001	−2.653	−2.982	− 2.324	< 0.0001
Level of life satisfaction	1 more score value	1.308	1.232	1.385	< 0.0001	1.122	1.045	1.198	< 0.0001
Physical Component Summary (PCS)	1 more PCS score value	−0.026	− 0.048	− 0.004	0.022	− 0.089	− 0.110	− 0.068	< 0.0001
Length of stay (years)	<=9	0	–	–	–	0	–	–	–
	> = 10	−0.388	− 0.639	− 0.138	0.002	− 0.719	− 0.966	−0.472	< 0.0001
Age group (years)	15–39	0	–	–	–	0	–	–	–
	40–64	−0.914	−1.165	−0.663	< 0.0001	− 0.618	− 0.864	−0.371	< 0.0001
Sex	Male	0	–	–	–	0	–	–	–
	Female	−0.705	− 0.955	− 0.455	< 0.0001	− 0.726	−0.975	− 0.477	< 0.0001
Education level	High	0	–	–	–	0	–	–	–
	Middle/Low	−0.308	−0.565	− 0.051	0.019	0.225	−0.022	0.471	0.074
Employment status	Employed	0	–	–	–	0	–	–	–
	Formerly employed	−1.948	−2.281	−1.616	< 0.0001	−1.502	− 1.821	− 1.184	< 0.0001
Area of origin	Europe	0	–	–	–	0	–	–	–
	North Africa	−0.597	−0.988	− 0.206	0.003	− 0.079	−0.458	0.300	0.684
	Sub-Saharan Africa	−0.136	−0.682	0.410	0.625	0.611	0.094	1.129	0.021
	Central-western Asia	0.902	0.404	1.400	< 0.0001	0.979	0.506	1.452	< 0.0001
	East Asia / Pacific	1.158	0.656	1.661	< 0.0001	1.026	0.552	1.500	< 0.0001
	The Americas	−0.112	−0.618	0.395	0.666	−0.364	− 0.840	0.111	0.133

Figure 1 summarizes the conceptual path analysis, showing the coefficients, adjusted for confounders, of the direct relationship between exposure factor (S-PWD) and outcome (MCS) and their indirect relationship decomposed into relationships between S-PWD and each mediating factor (S-PL, LS, PCS) and between each mediating factor and MCS. S-PWD was negatively associated with MCS (β_3_:-1.780), PCS (β_1.3_:-1.107), and LS (β_1.2_:-0.463), while it was positively associated with S-PL (β_1.1_:0.113). S-PL (β_2.1_:-2.627) and PCS (β_2.3_:-0.089) were negatively associated with MCS, that was positively associated with LS (β_2.2_:1.116).

Table 3 shows the results from the path analysis, which decomposed the total effect of workplace discrimination on MCS into direct and indirect effects (the full path analysis output is shown in Additional file 1). The direct effect of S-PWD on MCS accounted for 71.3% of the total (β:-1.78 out of − 2.49). The proportion of total effect mediated by psychophysical factors was 28.7%, of which 11.9% was attributable to S-PL (indirect effect β_1.1_*β_2.1_:-0.30), 20.7% to LS (indirect effect β_1.2_*β_2.2_:-0.52), and 3.9% to PCS (indirect effect β_1.3_*β_2.3_:0.10).

**Table 3:** Path coefficients and proportion (*100) of effects of self-perceived workplace discrimination (S-PWD) on mental component summary (MCS) mediated by psychophysical factors. Results from path analysis model. SCIF survey 2011–2012

Effects	Path coefficients		Proportion of total effect (calculated by model coefficients) mediated by each factor	Proportion of total effect (absolute values of model coefficients) mediated by each factor
	Estimate	95%CI	%	%
Direct of S-PWD on MCS	*−1.78*	*−2.09; −1.47*	*71.3*	*63.4*
Indirect of S-PWD on MCS	*−0.72*	*−0.83; − 0.61*	*28.7*	*36.6*
*Indirect of S-PWD mediated by self-perceived loneliness on MCS*	*−0.30*	*−0.36; − 0.24*	*11.9*	*11.9*
*Indirect of S-PWD mediated by level of life satisfaction on MCS*	*−0.52*	*− 0.61; − 0.43*	*20.7*	*20.7*
*Indirect of S-PWD mediated by Physical Component Summary on MCS*	*0.10*	*0.07; 0.13*	*−3.9*	*3.9*
Total of S-PWD on MCS	*−2.49*	*−2.82; − 2.17*	*100*	*100*

In the path analysis, the likelihood-ratio test was LR = 1523.14 (number of degrees of freedom equal to k = 21), with the *p-*value (χ^2^_k_ ≥ LR) < 0.001; this result indicates that the model with all predictors fit significantly better than the model with only the intercept.

## Discussion

This study investigated whether self-perceived workplace discrimination has any role in the mental health status of immigrants living and working in Italy, taking into consideration other personal experiences like self-perceived loneliness, level of life satisfaction, and perceived physical health.

We hypothesized that S-PWD may affect MCS directly as well as through the influences of some psychophysical factors, personal experiences (e.g. S-PL and LS), and self-reported physical status. Our results underline and quantify the relationship between S-PWD and mental health outcomes, directly as well as through S-PL, LS, and PCS as mediators.

In our study S-PWD seemed to act on MCS both through a direct relationship, which we estimated as 68.9% of the total effect, and through an indirect relationship mediated by S-PL (11.9%), LS (20.7%), and PCS (3.9%). In particular, we observed a negative effect of S-PWD on MCS when it was mediated by S-PL and LS, while the indirect effect mediated by PCS was positive, as the product of two negative effects (S-PWD on PCS and PCS on MCS).

Our findings appear to support previous research that underlined the relationship between workplace discrimination and mental health in a large, heterogeneous immigrant sample [17]. It would seem that self-perceived discrimination – whether suffered during a current or past job – can act as a predictor of alterations in self-perceived mental health, as already demonstrated by a number of other studies. In particular, perceived discrimination has been associated with mental health conditions such as anxiety, depression, fear, frustration, helplessness, hopelessness, paranoia, resentment, and low self-esteem [12, 17, 20, 36, 44, 45].

Psychosocial risk factors, such as anxiety, insecurity, low self-esteem, social isolation, and the lack of control over work and home life increase the risk of poor mental and physical health. The lower people are in the social hierarchy of industrialized countries, the more common these problems become. In the case of immigrant workers self-reporting discrimination, for whom we can hypothesize long-term stress, these individuals become more vulnerable to a wide range of poor health conditions, acting as an accelerator of mental distress [18, 36, 46, 47].

An explanation of this process can be found in the construction vs. deconstruction of professional and personal life projects of migrants [48]. In fact, the process of immigration itself constitutes a pool of life projects and expectations of those people who decide to change their life. The status of these projects can thus greatly influence overall life satisfaction. Among migrants, these projects are usually work-oriented, devised and implemented to guarantee economic survival, obtain personal and professional satisfaction, obtain rights connected with having a residence permit, and improve social inclusion by becoming part of the host country [49, 50].

Discrimination in the workplace can be extremely harmful, especially for immigrant populations, given that work (and its implications) is one of their priority objectives [17, 25, 51, 52].

Overall, the literature shows that perceived discrimination in the workplace is a significant stressor for all population groups because one’s job represents a strong link with society; it is an important way to feel part of this new world [12, 47]. Experiences of unfair treatment and daily difficulties for any reason can therefore have an impact on mental and physical health [12, 23, 25].

The workplace is a social context where discrimination is experienced due to limited access to certain types of jobs, bad relationships between workers and management, or to the characteristics of the job itself [45]. Not being valued and respected in the workplace, imbalanced job design, and occupational uncertainty may negatively affect mental health, as can interacting with individual personality characteristics, attitudes, and coping [20].

Experiences of perceived discrimination may vary in relation to many contextual factors as well as to other personal and economic resources. Immigrants who experienced discrimination were most likely to report worsening self-reported mental health, with a higher risk of feelings of sadness, depression, and loneliness [3, 6, 7, 11].

Our findings suggest that the low life satisfaction and perception of loneliness self-reported by immigrants in Italy could have a negative effect on their mental health status. Indeed, our results seem to support the hypothesis that discriminatory experiences may affect mental health through stress responses, which explain part of the effect on MCS of exposure to S-PWD, as suggested by the indirect negative effect of loneliness and low life satisfaction on MCS.

In our study, immigrants in almost all cases reported ethnic/cultural-related factors as the cause of their experience of discrimination in the workplace: being a foreigner, not speaking Italian well, skin colour, religion. It is interesting to underline that, unlike some other European countries, for example France or the United Kingdom, Italy has not experienced immigration from former colonies, with immigrants speaking the same language as in the host country, which means that integration may be even more difficult. In different countries, empirical evidence indicates a negative relationship between perceived ethnic discrimination and life satisfaction or sense of loneliness [53].

Immigrants face the integration process with an inner sense of inadequacy with regard to the host country and the dominant culture. Losing one’s job or perceiving discrimination could generate a deep sense of self-isolation, perceived social exclusion, and low sense of self-efficacy. Inevitably, this could affect the well-being or mental health status of these persons [7, 51].

Our findings of worse perceived mental health in people who had been in Italy longer than 10 years was similar to the findings of previous Canadian studies, which showed poorer mental health status among long-term immigrants than among recent immigrants [54, 55]. It is interesting to note that in a previous study conducted in Italy, psychotic disorders were more frequently diagnosed in immigrants who had had a residence permit for a long time, i.e. those who had been living in the country longer, than in those who had been living in the country for a shorter amount of time [56].

We also found that having lost one’s job (and therefore being unemployed at the time of the interview) may have negatively affected good mental health status among immigrants. In general, immigrants in a host country have invested considerably in personal projects [51]. Achieving medium- or long-term personal goals is an important factor in their improved life-satisfaction [53]. The literature shows that immigrants expect to be recognised as people who contribute to the receiving society in terms of experience and resources as well as in social and cultural wealth. They also expect their rights as citizens to be recognised at least to the degree that they were in their native country [57]. A collapse of these expectations, such as job loss, could negatively affect their life satisfaction in the host country [51, 53].

### Strengths and limitations

The strength of this study is that it was conducted in a large sample of Italian immigrants, where the first generation still makes up most of the foreign population, which has been strongly affected by the economic crisis and which has been subjected to a concerning increase in xenophobic episodes. Further, because its geographical position makes it the most common port of entry to Europe and thus migration here has very specific and unique characteristics, Italy is the ideal setting to study immigrants in terms of their mental health as well. Moreover, to the best of our knowledge, there have been few studies in Southern Europe that have investigated the implications of perceived discrimination in the workplace [12, 35, 36, 58].

Our study also extends existing research by examining the independent effects of mental health on other factors strictly related to the perception of one’s life condition. In particular, we considered the potential role of some personal experiences (loneliness, level of life satisfaction) and self-perceived physical health in the association between discrimination and self-perceived mental health.

One possible limitation of this study is that cross-sectional data can make it difficult to discern causality in the association observed. However, theoretical perspectives support the idea that perceived discrimination adversely affects mental health outcomes [27].

Furthermore, this study relies exclusively on self-reporting, through variables measured by single questions, rather than on validated instruments. However, many of the current studies in this area involve perceptions of discriminatory treatment based on self-reporting of life events and personal experiences rather than on objectively observed discrimination [27]. Moreover, it has been demonstrated that self-perceived health is a reliable predictor of mortality [59], reason for which it has frequently been used as an outcome measure in numerous studies on immigrant health [60].

Another limitation is that we did not have any information about the time frame, the regularity of discrimination experience, whether or not the immigrants had experienced discrimination in other areas of life, or information about income, a factor that leaves immigrants vulnerable to discrimination and is also associated with factors such as life satisfaction and physical health.

Finally, our decision to dichotomize some variables (education level, age, and length of stay) to obtain more robust estimates and to make the interpretation easier, may have produced an information loss about collected data, albeit modest, at least for categorical variables.

## Conclusions

Our findings of a relationship between perceived workplace discrimination and mental health status – mediated by loneliness, life satisfaction, and perceived physical health – suggest that non-occupational personal and psychosocial factors may act as stressors for immigrant workers, who are the weakest link in the labour market in developed countries, particularly during a global recession [11, 29, 49, 51]. In Italy as well as in other European countries, the socioeconomic shock induced by the COVID-19 pandemic may represent a potential risk for the immigrant population of increasing experiences of discrimination, precarious working conditions, mistreatment, and racism, especially for those subjects at greater risk of social exclusion and marginalization. In addition, discrimination generated by the social stigma of being an immigrant continues to persist; immigrants are often seen as carriers of infectious diseases, a prejudice that has been accentuated during the COVID-19 pandemic. From this perspective our study can be considered an indication for an overall public health response based on the WHO’s Health in All Policies approach, which, although not designed specifically for migrant health, has an incontestable role in addressing social determinants of health. These policies, in addition to any workplace-based interventions, should be to facilitate the social integration of immigrants and their access to health services, in particular those dedicated to addressing mental health issues. In fact, a systematic review and meta-analysis on the effects of non-health-targeted policies documented increased risks of poor mental health in the presence of restricted access to welfare and health services [61]. Future efforts should focus on longitudinal prospective studies on immigrant workers that explore the mechanisms that mediate the effects of perceived discrimination on mental health.

## Supplementary Information

**Additional file 1.** Path analysis model of relation of S-PWD on MCS mediated by psychophysical factors.

## Data Availability

The data that support the findings of this study were made available by Istat, but restrictions apply to the availability of these data, which were used under license for the current study, and are thus not publicly available.

## Abbreviations

Istat: Italian National Institute of Statistics
LS: Life satisfaction
MCS: Mental Component Summary
PCS: Physical Component Summary
SCIF: Social Conditions and Integration of Foreign Citizens
S-PDW: Self-perceived discrimination in the workplace
S-PL: Self-perceived loneliness
