# Supplementary material for: Self-perceived workplace discrimination and mental health among immigrant workers in Italy: a cross-sectional study
Source: BMC Psychiatry. 2021 Feb 9;21:85. doi: 10.1186/s12888-021-03077-6 (PMC7871130; doi:10.1186/s12888-021-03077-6)
Supplement: Supplementary file 1 — Additional file 1. Path analysis model of relation of S-PWD on MCS mediated by psychophysical factors. [file 12888_2021_3077_MOESM1_ESM.docx]

| **Appendix 1 – Path analysis model of relation of S-PWD on MCS mediated by psychophysical factors** | | | | | | | |
| --- | --- | --- | --- | --- | --- | --- | --- |
|  | | | | | | | |
| Path analysis model  Number of observations = 12,408 | | | | | | | |
| Estimation method = maximum likelihood | | | | | | | |
| Log likelihood = -162668.9 | | | | | | | |
| **OIM (observed information matrix)** | | | | | | |  |
|  | Coef. | Std.Err | z | P>\|z\| | [95% Conf. Interval] | |  |
| **S-PWD** |  |  |  |  |  |  |  |
| employment status | 0.02001 | 0.00914 | 2.19 | 0.0290 | 0.00210 | 0.03792 |  |
| age | -0.00172 | 0.00707 | -0.24 | 0.8080 | -0.01558 | 0.01214 |  |
| sex | -0.02249 | 0.00708 | -3.18 | 0.0010 | -0.03636 | -0.00862 |  |
| education level | -0.00377 | 0.00712 | -0.53 | 0.5970 | -0.01772 | 0.01018 |  |
| length of stay | 0.00971 | 0.00713 | 1.36 | 0.1730 | -0.00426 | 0.02367 |  |
| area of origin | -0.01509 | 0.00719 | -2.10 | 0.0360 | -0.02918 | -0.00099 |  |
| _cons | 0.18558 | 0.02133 | 8.70 | 0.0000 | 0.14378 | 0.22739 |  |
| **LS** |  |  |  |  |  |  |  |
| S-PWD | -0.46339 | 0.03765 | -12.31 | 0.0000 | -0.53719 | -0.38959 |  |
| _cons | 7.64140 | 0.01566 | 488.00 | 0.0000 | 7.61071 | 7.67209 |  |
| **S-PL** |  |  |  |  |  |  |  |
| S-PWD | 0.11332 | 0.00871 | 13.01 | 0.0000 | 0.09625 | 0.13039 |  |
| _cons | 0.14344 | 0.00362 | 39.60 | 0.0000 | 0.13634 | 0.15054 |  |
| **PCS** |  |  |  |  |  |  |  |
| S-PWD | -1.10741 | 0.13345 | -8.30 | 0.0000 | -1.36897 | -0.84585 |  |
| _cons | 54.88146 | 0.05550 | 988.87 | 0.0000 | 54.77268 | 54.99023 |  |
| **MCS** |  |  |  |  |  |  |  |
| S-PWD | -1.77969 | 0.15968 | -11.15 | 0.0000 | -2.09266 | -1.46672 |  |
| LS | 1.11628 | 0.03896 | 28.66 | 0.0000 | 1.03993 | 1.19263 |  |
| int_cs1_cat | -2.62721 | 0.16763 | -15.67 | 0.0000 | -2.95576 | -2.29866 |  |
| PCS | -0.08900 | 0.01079 | -8.25 | 0.0000 | -0.11014 | -0.06786 |  |
| employment status | -1.57367 | 0.16181 | -9.73 | 0.0000 | -1.89081 | -1.25653 |  |
| age | -0.62952 | 0.12578 | -5.01 | 0.0000 | -0.87604 | -0.38300 |  |
| sex | -0.74304 | 0.12467 | -5.96 | 0.0000 | -0.98738 | -0.49870 |  |
| education level | 0.22073 | 0.12530 | 1.76 | 0.0780 | -0.02486 | 0.46631 |  |
| length of stay | -0.72124 | 0.12590 | -5.73 | 0.0000 | -0.96800 | -0.47447 |  |
| area of origin | -0.35788 | 0.12685 | -2.82 | 0.0050 | -0.60650 | -0.10925 |  |
| _cons | 55.49558 | 0.79972 | 69.39 | 0.0000 | 53.92816 | 57.06299 |  |
| var(e.S-PWD ) | 0.00181 |  |  |  | 0.13923 | 0.14633 |  |
| var(e.LS) | 0.03195 |  |  |  | 2.45431 | 2.57954 |  |
| var(e.int_cs1_cat) | 0.00171 |  |  |  | 0.13131 | 0.13801 |  |
| var(e.PCS) | 0.40130 |  |  |  | 30.83145 | 32.40467 |  |
| var(e.MCS) | 0.55894 |  |  |  | 42.94332 | 45.13456 |  |
| LR test model vs satured: | | chi2(21)= | 1523.14 |  | Prob> chi2 = | 0.0000 |  |
|  |  |  |  |  |  |  |  |
|  |  |  |  |  |  |  |  |
|  |  |  |  |  |  |  |  |
| **Direct effects** | | | | | | |  |
|  | **Coef.** | **Std.Err** | **z** | **P>\|z\|** | **[95% Conf. Interval]** | |  |
| **S-PWD** | | | | | | |  |
| employment status | 0.02001 | 0.00914 | 2.19 | 0.0290 | 0.00210 | 0.03792 |  |
| age | -0.00172 | 0.00707 | -0.24 | 0.8080 | -0.01558 | 0.01214 |  |
| sex | -0.02249 | 0.00708 | -3.18 | 0.0010 | -0.03636 | -0.00862 |  |
| education level | -0.00377 | 0.00712 | -0.53 | 0.5970 | -0.01772 | 0.01018 |  |
| length of stay | 0.00971 | 0.00713 | 1.36 | 0.1730 | -0.00426 | 0.02367 |  |
| area of origin | -0.01509 | 0.00719 | -2.10 | 0.0360 | -0.02918 | -0.00099 |  |
| **LS** | | | | | | |  |
| S-PWD | -0.46339 | 0.03765 | -12.31 | 0.0000 | -0.53719 | -0.38959 |  |
| employment status | 0 | no path | | | | |  |
| age | 0 | no path | | | | |  |
| sex | 0 | no path | | | | |  |
| education level | 0 | no path | | | | |  |
| length of stay | 0 | no path | | | | |  |
| area of origin | 0 | no path | | | | |  |
| **S-PL** | | | | | | |  |
| S-PWD | 0.11332 | 0.00871 | 13.01 | 0.0000 | 0.09625 | 0.13039 |  |
| employment status | 0 | no path | | | | |  |
| age | 0 | no path | | | | |  |
| sex | 0 | no path | | | | |  |
| education level | 0 | no path | | | | |  |
| length of stay | 0 | no path | | | | |  |
| area of origin | 0 | no path | | | | |  |
| **PCS** | | | | | | |  |
| S-PWD | -1.10741 | 0.13345 | -8.30 | 0.0000 | -1.36897 | -0.84585 |  |
| employment status | 0 | no path | | | | |  |
| age | 0 | no path | | | | |  |
| sex | 0 | no path | | | | |  |
| education level | 0 | no path | | | | |  |
| length of stay | 0 | no path | | | | |  |
| area of origin | 0 | no path | | | | |  |
| **MCS** | | | | | | |  |
| S-PWD | -1.77969 | 0.15968 | -11.15 | 0.0000 | -2.09266 | -1.46672 |  |
| LS | 1.11628 | 0.03896 | 28.66 | 0.0000 | 1.03993 | 1.19263 |  |
| S-PL | -2.62721 | 0.16763 | -15.67 | 0.0000 | -2.95576 | -2.29866 |  |
| PCS | -0.08900 | 0.01079 | -8.25 | 0.0000 | -0.11014 | -0.06786 |  |
| employment status | -1.57367 | 0.16181 | -9.73 | 0.0000 | -1.89081 | -1.25653 |  |
| age | -0.62952 | 0.12578 | -5.01 | 0.0000 | -0.87604 | -0.38300 |  |
| sex | -0.74304 | 0.12467 | -5.96 | 0.0000 | -0.98738 | -0.49870 |  |
| education level | 0.22073 | 0.12530 | 1.76 | 0.0780 | -0.02486 | 0.46631 |  |
| length of stay | -0.72124 | 0.12590 | -5.73 | 0.0000 | -0.96800 | -0.47447 |  |
| area of origin | -0.35788 | 0.12685 | -2.82 | 0.0050 | -0.60650 | -0.10925 |  |
|  |  |  |  |  |  |  |  |
|  |  |  |  |  |  |  |  |
|  |  |  |  |  |  | |  |
| **Indirect effects** | | | | | | |  |
|  | **Coef.** | **Std.Err** | **z** | **P>\|z\|** | **[95% Conf. Interval]** | |  |
| **S-PWD** | | | | | | |  |
| employment status | 0 | no path | | | | |  |
| age | 0 | no path | | | | |  |
| sex | 0 | no path | | | | |  |
| education level | 0 | no path | | | | |  |
| length of stay | 0 | no path | | | | |  |
| area of origin | 0 | no path | | | | |  |
| **LS** | | | | | | |  |
| S-PWD | 0 | no path | | | | |  |
| employment status | -0.00927 | 0.00430 | -2.16 | 0.0310 | -0.01770 | -0.00084 |  |
| age | 0.00080 | 0.00328 | 0.24 | 0.8080 | -0.00563 | 0.00722 |  |
| sex | 0.01042 | 0.00339 | 3.08 | 0.0020 | 0.00378 | 0.01706 |  |
| education level | 0.00175 | 0.00330 | 0.53 | 0.5970 | -0.00472 | 0.00822 |  |
| length of stay | -0.00450 | 0.00332 | -1.35 | 0.1760 | -0.01101 | 0.00202 |  |
| area of origin | 0.00699 | 0.00338 | 2.07 | 0.0390 | 0.00037 | 0.01362 |  |
| **S-PL** | | | | | | |  |
| S-PWD | 0 | no path | | | | |  |
| employment status | 0.00227 | 0.00105 | 2.16 | 0.0310 | 0.00021 | 0.00433 |  |
| age | -0.00020 | 0.00080 | -0.24 | 0.8080 | -0.00177 | 0.00138 |  |
| sex | -0.00255 | 0.00083 | -3.09 | 0.0020 | -0.00417 | -0.00093 |  |
| education level | -0.00043 | 0.00081 | -0.53 | 0.5970 | -0.00201 | 0.00116 |  |
| length of stay | 0.00110 | 0.00081 | 1.35 | 0.1760 | -0.00049 | 0.00269 |  |
| area of origin | -0.00171 | 0.00083 | -2.07 | 0.0380 | -0.00333 | -0.00009 |  |
| **PCS** | | | | | | |  |
| S-PWD | 0 | no path | | | | |  |
| employment status | -0.02216 | 0.01047 | -2.12 | 0.0340 | -0.04267 | -0.00165 |  |
| age | 0.00191 | 0.00783 | 0.24 | 0.8080 | -0.01345 | 0.01726 |  |
| sex | 0.02491 | 0.00839 | 2.97 | 0.0030 | 0.00846 | 0.04136 |  |
| education level | 0.00417 | 0.00790 | 0.53 | 0.5970 | -0.01131 | 0.01966 |  |
| length of stay | -0.01075 | 0.00800 | -1.34 | 0.1790 | -0.02642 | 0.00493 |  |
| area of origin | 0.01671 | 0.00821 | 2.03 | 0.0420 | 0.00061 | 0.03281 |  |
| **MCS** | | | | | | |  |
| S-PWD | -0.71641 | 0.05552 | -12.90 | 0.0000 | -0.82524 | -0.60759 |  |
| LS | 0 | no path | | | | |  |
| S-PL | 0 | no path | | | | |  |
| PCS | 0 | no path | | | | |  |
| employment status | -0.04995 | 0.02305 | -2.17 | 0.0300 | -0.09512 | -0.00478 |  |
| age | 0.00430 | 0.01765 | 0.24 | 0.8080 | -0.03030 | 0.03889 |  |
| sex | 0.05614 | 0.01806 | 3.11 | 0.0020 | 0.02075 | 0.09153 |  |
| education level | 0.00941 | 0.01778 | 0.53 | 0.5970 | -0.02544 | 0.04425 |  |
| length of stay | -0.02422 | 0.01786 | -1.36 | 0.1750 | -0.05923 | 0.01078 |  |
| area of origin | 0.03766 | 0.01812 | 2.08 | 0.0380 | 0.00214 | 0.07318 |  |
|  |  |  |  |  |  |  |  |
|  |  |  |  |  |  |  |  |
| ***Total effects*** | | | | | | |  |
|  | **Coef.** | **Std.Err** | **z** | **P>\|z\|** | **[95% Conf. Interval]** | |  |
| **S-PWD** | | | | | | |  |
| employment status | 0.02001 | 0.00914 | 2.19 | 0.0290 | 0.00210 | 0.03792 |  |
| age | -0.00172 | 0.00707 | -0.24 | 0.8080 | -0.01558 | 0.01214 |  |
| sex | -0.02249 | 0.00708 | -3.18 | 0.0010 | -0.03636 | -0.00862 |  |
| education level | -0.00377 | 0.00712 | -0.53 | 0.5970 | -0.01772 | 0.01018 |  |
| length of stay | 0.00971 | 0.00713 | 1.36 | 0.1730 | -0.00426 | 0.02367 |  |
| area of origin | -0.01509 | 0.00719 | -2.10 | 0.0360 | -0.02918 | -0.00099 |  |
| **LS** | | | | | | |  |
| S-PWD | -0.46339 | 0.03765 | -12.31 | 0.0000 | -0.53719 | -0.38959 |  |
| employment status | -0.00927 | 0.00430 | -2.16 | 0.0310 | -0.01770 | -0.00084 |  |
| age | 0.00080 | 0.00328 | 0.24 | 0.8080 | -0.00563 | 0.00722 |  |
| sex | 0.01042 | 0.00339 | 3.08 | 0.0020 | 0.00378 | 0.01706 |  |
| education level | 0.00175 | 0.00330 | 0.53 | 0.5970 | -0.00472 | 0.00822 |  |
| length of stay | -0.00450 | 0.00332 | -1.35 | 0.1760 | -0.01101 | 0.00202 |  |
| area of origin | 0.00699 | 0.00338 | 2.07 | 0.0390 | 0.00037 | 0.01362 |  |
| **S-PL** | | | | | | |  |
| S-PWD | 0.11332 | 0.00871 | 13.01 | 0.0000 | 0.09625 | 0.13039 |  |
| employment status | 0.00227 | 0.00105 | 2.16 | 0.0310 | 0.00021 | 0.00433 |  |
| age | -0.00020 | 0.00080 | -0.24 | 0.8080 | -0.00177 | 0.00138 |  |
| sex | -0.00255 | 0.00083 | -3.09 | 0.0020 | -0.00417 | -0.00093 |  |
| education level | -0.00043 | 0.00081 | -0.53 | 0.5970 | -0.00201 | 0.00116 |  |
| length of stay | 0.00110 | 0.00081 | 1.35 | 0.1760 | -0.00049 | 0.00269 |  |
| area of origin | -0.00171 | 0.00083 | -2.07 | 0.0380 | -0.00333 | -0.00009 |  |
| **PCS** | | | | | | |  |
| S-PWD | -1.10741 | 0.13345 | -8.30 | 0.0000 | -1.36897 | -0.84585 |  |
| employment status | -0.02216 | 0.01047 | -2.12 | 0.0340 | -0.04267 | -0.00165 |  |
| age | 0.00191 | 0.00783 | 0.24 | 0.8080 | -0.01345 | 0.01726 |  |
| sex | 0.02491 | 0.00839 | 2.97 | 0.0030 | 0.00846 | 0.04136 |  |
| education level | 0.00417 | 0.00790 | 0.53 | 0.5970 | -0.01131 | 0.01966 |  |
| length of stay | -0.01075 | 0.00800 | -1.34 | 0.1790 | -0.02642 | 0.00493 |  |
| area of origin | 0.01671 | 0.00821 | 2.03 | 0.0420 | 0.00061 | 0.03281 |  |
| **MCS** | | | | | | |  |
| S-PWD | -2.49610 | 0.16519 | -15.11 | 0.0000 | -2.81987 | -2.17233 |  |
| LS | 1.11628 | 0.03896 | 28.66 | 0.0000 | 1.03993 | 1.19263 |  |
| S-PL | -2.62721 | 0.16763 | -15.67 | 0.0000 | -2.95576 | -2.29866 |  |
| PCS | -0.08900 | 0.01079 | -8.25 | 0.0000 | -0.11014 | -0.06786 |  |
| employment status | -1.62362 | 0.16338 | -9.94 | 0.0000 | -1.94384 | -1.30340 |  |
| age | -0.62523 | 0.12701 | -4.92 | 0.0000 | -0.87416 | -0.37629 |  |
| sex | -0.68690 | 0.12587 | -5.46 | 0.0000 | -0.93359 | -0.44021 |  |
| education level | 0.23014 | 0.12655 | 1.82 | 0.0690 | -0.01790 | 0.47817 |  |
| length of stay | -0.74546 | 0.12714 | -5.86 | 0.0000 | -0.99466 | -0.49626 |  |
| area of origin | -0.32022 | 0.12810 | -2.50 | 0.0120 | -0.57128 | -0.06916 |  |
